# Correspondence: Isometric hip strength impairments in patients with hip dysplasia are improved but not normalized 1 year after periacetabular osteotomy: a cohort study of 82 patients

**DOI:** 10.1080/17453674.2021.1982480

**Published:** 2021-10-05

**Authors:** Jacobsen Julie Sandell, Jakobsen Stig Storgaard, Søballe Kjeld, Hölmich Per, Thorborg Kristian, Mingjin Zhong, Weimin Zhu

**Affiliations:** Research Centre for Health and Welfare Technology, Programme for Rehabilitation, VIA University College, Aarhus; Research Unit for General Practice in Aarhus, Aarhus.; Department of Orthopaedic Surgery, Aarhus University Hospital, Aarhus.; Department of Clinical Medicine, Aarhus University, Aarhus.; Sports Orthopaedic Research Center-Copenhagen (SORCC), Department of Orthopaedic Surgery, Copenhagen University Hospital, Hvidovre.; Physical Medicine and Rehabilitation Research-Copenhagen (PMR-C), Department of Physical and Occupational Therapy, Copenhagen University Hospital, Hvidovre, Denmark.; Department of Sports Medicine, The First Affiliated Hospital of Shenzhen University, Health Science Center, Shenzhen Second People’s Hospital, Shenzhen, Guangdong, China

Jacobsen et al. Acta Orthop 2021; 92(3): 285-291. DOI 10.1080/17453674.2020.1864911

*Sir,—*We read with great interest the article “Isometric hip strength impairments in patients with hip dysplasia are improved but not normalized 1 year after periacetabular osteotomy: a cohort study of 82 patients” by Jacobsen et al. (2021).

The authors investigated isometric hip muscle strength in patients with hip dysplasia, before and 1 year after periacetabular osteotomy, and compared this with healthy volunteers. We agree with the conclusions drawn by the authors. However, there are some issues we like to comment on.

First, healthy volunteers did not undergo any imaging, and it is therefore unknown if they were radiologically healthy despite having no hip symptoms or other joint abnormalities. Furthermore, the findings of positive FADIR test (3/50) and positive FABER test (2/50) in some healthy volunteers could be related to persons not being “healthy.” Hip disorders should be diagnosed based on a combination of symptoms, clinical signs and imaging findings. Hence, it is currently unknown whether asymptomatic hips have hidden other hip disorders. Consequently, we believe that healthy people should be evaluated more comprehensively.

Second, 89 patients with bilateral hip dysplasia were recruited in this study. However, inclusion of bilateral hip dysplasia patients may also lead to bias. Previous studies showed that the hip muscle strength of the contralateral hip joint in patients with unilateral femoroacetabular impingement syndrome (Malloy et al. 2019) or hip osteoarthritis (Arokoski et al. 2002, Diamond et al. 2016) will also be affected. We firmly believe that this condition can also occur in patients with hip dysplasia. However, side differences of hip muscle strength between affected and contralateral leg were not analyzed bilaterally in patients affected with hip dysplasia.

Third, hand-held instead of stabilized dynamometry which may be less robust than other forms of strength testing (e.g., Biodex system) was used for strength assessment of hip flexion, extension, abduction, and adduction. Intrarater reliability of hand-held dynamometry has been shown to be lower compared with stabilized dynamometry due to the influence of the investigator’s strength to resist the measured forces (Thorborg et al. 2009, Casartelli et al. 2010).

Furthermore, the considerable hip dysplasia-related hip muscle weakness observed pre- and post-operation in this study could potentially originate from different factors: a mechanical/anatomical limit, qualitative (fatty degeneration), quantitative (atrophy) morphologic alterations or reduced muscle activation (possibly related to pain and/or fear of pain) during isometric hip muscle contraction. Although the authors found that an increased hip muscle strength was associated with higher hip functional scores, however, finding that hip muscle weakness was predictive of hip–specific outcomes does not imply causality. It is possible that poor surgical outcomes caused disuse muscle weakness that are then detected through dynamometer rather than hip muscle weakness affecting patient clinical outcomes. Further research would be necessary to clarify this point. Additionally, the cross sectional design did not clarify the development in muscle strength over time in patients with hip dysplasia and the hand-held dynamometer can’t indicate which specific muscles of the hip is weakened. We do not know which specific muscles of hip have weakened and which have not after periacetabular osteotomy. However, hip muscle size can be assessed by quantifying the cross-sectional area using axial cuts of pelvis magnetic resonance imaging (Malloy et al. 2019, Gao et al. 2021).

**Mingjin Zhong and Weimin Zhu**

*Department of Sports Medicine, The First Affiliated Hospital of Shenzhen University, Health Science Center, Shenzhen Second People’s Hospital, Shenzhen, Guangdong, China*

*Email:* sportsmedzhong@sina.com

*Sir,—* We are pleased that Dr. Zhong agrees with the conclusion of our findings which states 1) “Isometric hip muscle strength is impaired in patients with symptomatic dysplastic hips measured before PAO”, and 2) “1 year after surgery, isometric hip flexion and abduction strength had improved but muscle strength did not reach that of healthy volunteers.” Furthermore, we would also like to thank Dr. Zhong for the possibility to elaborate on key issues raised in the letter, described point by point.

Point 1: The healthy volunteers were asymptomatic and excluded in case of pain, comorbidity, previous trauma or surgery. Radiology was used only in the patient population as it was considered unethical to expose the asymptomatic population to radiation. Therefore, “asymptomatic” would have been a better term than “healthy” volunteers. Regarding imaging findings, we agree that imaging alone cannot be used to determine whether (or not) participants are healthy. Instead a combination of symptoms, clinical signs and imaging should be used to assess the presence of “hip disease”, as agreed for femoroacetabular impingement syndrome (Griffin et al. 2016). This is why the findings of positive FADIR/FABER tests do not indicate whether participants are healthy. Painful FADIR tests have been documented in 12–15% of asymptomatic participants (Czuppon et al. 2017); this is most likely due to the high sensitivity and false positive rate of this test (Reiman et al. 2013). We consider the FADIR test to be positive only if it replicates known symptoms (Troelsen et al. 2009). Therefore, in the asymptomatic volunteers, it would have been less confusing if we had described whether a test was painful instead of labeling the test as positive or negative. In the case of missing “hidden” pathology in the asymptomatic volunteers, the muscle strength deficit seen in the patients in our study might have been larger, but this would not have changed our conclusion that “isometric hip muscle strength is impaired in patients with symptomatic dysplastic hips.”

Point 2: In our sample 63% had bilateral symptoms (89% radiological bilateral affection). The patients had a significant strength deficit of both symptomatic and asymptomatic side compared to asymptomatic volunteers, and apart from hip abduction, there was no statistically significant differences between the two, as expected, and as correctly pointed out by Dr. Zhong (Table, new data). Moreover, the majority of patients with hip dysplasia are bilaterally affected, and therefore we do not consider side comparison relevant as most often the contralateral side cannot be considered “normal.”

**Table:** Mean hip muscle strength (Nm/kg) in 50 asymptomatic volunteers and 37 patients with unilateral symptoms related to hip dysplasia divided in asymptomatic and symptomatic hips

	Asymptomatic hip (volunteers)		Asymptomatic hip (dysplasia ptts.)		Symptomatic hip (dysplasia ptts.)		Difference **^a^** (95% CI)	p-value	Difference ^b^ (95% CI)	p-value	Difference **^c^** (95% CI)	p-value
	n	Mean (SD)	n	Mean (SD)	n	Mean (SD)						
Flexion	50	1.8 (0.34)	37	1.2 (0.40)	37	1.2 (0.35)	0.60 (0.44–0.76)	< 0.001	0.60 (0.45–0.75)	< 0.001	0.00 (-0.17 to 0.17)	1.0
Extension	50	2.5 (0.63)	36	1.9 (0.68)	36	1.8 (0.59)	0.60 (0.32–0.88)	0.001	0.70 (0.43–0.97)	< 0.001	0.10 (-0.20 to 0.40)	0.5
Abduction	50	1.5 (0.37)	36	1.3 (0.35)	37	1.1 (0.38)	0.20 (0.04–0.36)	0.01	0.40 (0.24–0.56)	< 0.001	0.20 (0.31 to 0.37)	0.02
Adduction	50	1.5 (0.46)	36	1.1 (0.37)	37	1.0 (0.31)	0.40 (0.22–0.58)	< .001	0.50 (0.33–0.67)	< 0.001	0.10 (-0.06 to 0.26)	0.2

a Difference in muscle strength between hip in asymptomatic volunteers compared with asymptomatic hip in hip dysplasia patients (ptts.) with unilateral hjp symptoms.

b Difference in hip muscle strength between hip in asymptomatic volunteers compared with symptomatic hip in hip dysplasia patients with unilateral hip symptoms.

c Difference in hip muscle strength between hip in asymptomatic and symptomatic hip in hip dysplasia patients with unilateral hip symptoms.

Point 3: Dr. Zhong stated that the intra-rater reliability of hand-held dynamometry (HHD) was worse compared with stabilized dynamometry. This statement is not supported by the studies referred to in the letter (Thorborg et al. 2010, Casartelli et al. 2011) or in previous studies (Thorborg et al. 2010, 2013, Casartelli et al. 2011, Kemp et al. 2013, Chamorro et al. 2017). On the contrary, it has been shown that the reliability of HHD compared with isokinetic dynamometry was not inferior but comparable (Chamorro et al. 2017). On this basis, we consider HHD applicable to evaluate muscle strength in this population, where muscle strength of the tester seems to surpass the strength of the person being tested.

Finally, our study was not designed to investigate whether the reported strength deficit originated from either mechanical, qualitative or quantitative causes. Instead, muscle strength was tested with a HHD in 4 directions (Thorborg et al. 2010), and therefore deficits of specific muscles were not investigated. Nevertheless, we consider knowledge of deficits in specific directions useful in clinical practice since this knowledge can support monitoring of weakness and direct exercise interventions (Kemp et al. 2019).

**Julie Sandell Jacobsen ^1,2^, Stig Storgaard Jakobsen ^3^, Kjeld Søballe ^3,4^, Per Hölmich ^5^, Kristian Thorborg ^5,6^**

*^1^Research Centre for Health and Welfare Technology, Programme for Rehabilitation, VIA University College, Aarhus.*

*^2^Research Unit for General Practice in Aarhus, Aarhus.*

*^3^Department of Orthopaedic Surgery, Aarhus University Hospital, Aarhus.*

*^4^Department of Clinical Medicine, Aarhus University, Aarhus.*

*^5^Sports Orthopaedic Research Center-Copenhagen (SORC-C), Department of Orthopaedic Surgery, Copenhagen University Hospital, Hvidovre.*

*^6^Physical Medicine and Rehabilitation Research-Copenhagen (PMR-C), Department of Physical and Occupational Therapy, Copenhagen University Hospital, Hvidovre, Denmark.*

*Correspondence:* jsaj@via.dk

## References

[CIT0001] Arokoski M H, Arokoski J P, Haara M, Kankaanpää M, Vesterinen M, Niemitukia L H, Helminen H J. Hip muscle strength and muscle cross sectional area in men with and without hip osteoarthritis. J Rheumatol 2002; 29: 2185–95. 12375331

[CIT0002] Casartelli N C, Maffiuletti N A, Item-Glatthorn J F, Staehli S, Bizzini M, Impellizzeri F M, Leunig M. Hip muscle weakness in patients with symptomatic femoroacetabular impingement. Osteoarthritis Cartilage 2011; 19: 816–21. 21515390 10.1016/j.joca.2011.04.001

[CIT0003] Chamorro C, Armijo-Olivo S, De la Fuente C, Fuentes J, Javier Chirosa L. Absolute reliability and concurrent validity of hand held dynamometry and isokinetic dynamometry in the hip, knee and ankle joint: systematic review and meta-analysis. Open Med (Wars) 2017; 12: 359–75. 29071305 10.1515/med-2017-0052 PMC5651404

[CIT0004] Czuppon S, Prather H, Hunt D M, Steger-May K, Bloom N J, Clohisy J C, Larsen R, Harris-Hayes M. Gender-dependent differences in hip range of motion and impingement testing in asymptomatic college freshman athletes. PM R 2017; 9(7): 660–7. 27840297 10.1016/j.pmrj.2016.10.022 PMC5425325

[CIT0005] Diamond L E, Wrigley T V, Hinman R S, Hodges P W, O’Donnell J, Takla A, Bennell K L. Isometric and isokinetic hip strength and agonist/antagonist ratios in symptomatic femoroacetabular impingement. J Sci Med Sport 2016; 19: 696–701. 26526760 10.1016/j.jsams.2015.10.002

[CIT0006] Gao Y, Lyu X, Liu Q, Meng Y, Wang J, Pan S. Quantitative evaluation of hip muscle atrophy in patients with unilateral slipped capital femoral epiphysis based on magnetic resonance imaging. Acad Radiol 2021; 28(8): 1125–32. 32540199 10.1016/j.acra.2020.05.007

[CIT0007] Griffin D R, Dickenson E J, O’Donnell J, Agricola R, Awan T, Beck M, Clohisy J C, Dijkstra H P, Falvey E, Gimpel M, Hinman R S, Hölmich P, Kassarjian A, Martin H D, Martin R, Mather R C, Philippon M J, Reiman M P, Takla A, Thorborg K, Walker S, Weir A, Bennell K L. The Warwick Agreement on femoroacetabular impingement syndrome (FAI syndrome): An international consensus statement. Br J Sports Med 2016; 50(19): 1169–76. 27629403 10.1136/bjsports-2016-096743

[CIT0008] Jacobsen J S, Jakobsen S S, Søballe K, Hölmich P, Thorborg K. Isometric hip strength impairments in patients with hip dysplasia are improved but not normalized 1 year after periacetabular osteotomy: a cohort study of 82 patients. Acta Orthop 2021; 92(3): 285–91. 33538223 10.1080/17453674.2020.1864911 PMC8231359

[CIT0009] Kemp J L, Schache A G, Makdissi M, Sims K J, Crossley K M. Greater understanding of normal hip physical function may guide clinicians in providing targeted rehabilitation programmes. J Sci Med Sport 2013; 16(4): 292–6. 23266242 10.1016/j.jsams.2012.11.887

[CIT0010] Kemp J L, King M G, Barton C, Schache A G, Thorborg K, Roos E M, Scholes M, Grimaldi A, Semciw A I, Freke M, Risberg M A, Reiman M P, Mayes S, Pizzari T, Heerey J J, Lawrenson P R, Ingelsrud L H H, Crossley K M. Is exercise therapy for femoroacetabular impingement in or out of FASHIoN? We need to talk about current best practice for the non-surgical management of FAI syndrome. Br J Sports Med 2019; 53(19): 1204–5. 30626598 10.1136/bjsports-2018-100173

[CIT0011] Malloy P, Stone A V, Kunze K N, Neal W H, Beck E C, Nho S J. Patients with unilateral femoroacetabular impingement syndrome have asymmetrical hip muscle cross-sectional area and compensatory muscle changes associated with preoperative pain level. Arthroscopy 2019; 35: 1445–53. 30926193 10.1016/j.arthro.2018.11.053

[CIT0012] Reiman M P, Goode A P, Hegedus E J, Cook C E, Wright A A. Diagnostic accuracy of clinical tests of the hip: a systematic review with meta-analysis. Br J Sports Med 2013; 47(14): 893–902. 22773321 10.1136/bjsports-2012-091035

[CIT0013] Thorborg K, Petersen J, Magnusson S P, Hölmich P. Clinical assessment of hip strength using a hand-held dynamometer is reliable. Scand J Med Sci Sports 2010(3); 20: 493–501. 19558384 10.1111/j.1600-0838.2009.00958.x

[CIT0014] Thorborg K, Bandholm T, Hölmich P. Hip- and knee-strength assessments using a hand-held dynamometer with external belt-fixation are inter-tester reliable. Knee Surgery, Sport Traumatol Arthrosc 2013; 21(3): 550–5. 10.1007/s00167-012-2115-2 22773065

[CIT0015] Troelsen A, Mechlenburg I, Gelineck J, Bolvig L, Jacobsen S, Søballe K. What is the role of clinical tests and ultrasound in acetabular labral tear diagnostics? Acta Orthop 2009; 80(3): 314–8. 19421915 10.3109/17453670902988402 PMC2823204

